# ﻿*Monopyleglutinosa* (Gesneriaceae), a new species from the western slopes of the Ecuadorian Andes

**DOI:** 10.3897/phytokeys.210.89520

**Published:** 2022-09-28

**Authors:** John L. Clark, Franciso Tobar, Jeremy Keene

**Affiliations:** 1 Science Department, The Lawrenceville School, Lawrenceville, NJ 08648, USA The Lawrenceville School Lawrenceville United States of America; 2 Marie Selby Botanical Gardens, 811 South Palm Avenue, Sarasota, FL 34236, USA Marie Selby Botanical Gardens Sarasota United States of America; 3 Área de Investigación y Monitoreo de Avifauna, Aves y Conservación – BirdLife International, Quito, Ecuador Área de Investigación y Monitoreo de Avifauna Quito Ecuador; 4 Instituto Nacional de Biodiversidad, Herbario Nacional del Ecuador, Quito, Ecuador Instituto Nacional de Biodiversidad Quito Ecuador; 5 Science and Mathematics Department, Glenville State University, Glenville, WV 26351, USA Glenville State University Glenville United States of America

**Keywords:** Ecuador, Gesneriaceae, *Monopyle*, taxonomy

## Abstract

Exploratory field expeditions to the western slopes of the Ecuadorian Andes resulted in the discovery of a new species of *Monopyle* (Gesneriaceae). *Monopyleglutinosa* J.L.Clark & Keene, **sp. nov.** is described as a narrow endemic from lowland forests along the border of the Reserva Ecológica Los Illinizas in the Province of Cotopaxi. The new species is unique for the presence of glutinous or sticky trichomes on the calyx lobes and outer surface of the inferior ovary. Based on IUCN guidelines, a preliminary conservation status is assigned as Critically Endangered (CR).

## Abstract (es)

Las expediciones a los bosques de las laderas noroccidentales de los Andes de Ecuador dieron como resultado el descubrimiento de una nueva especie: *Monopyleglutinosa* J.L.Clark & Keene, **sp. nov.**, la cual es endémica de una reducida área en el borde de la Reserva Ecológica Los Illinizas en la provincia de Cotopaxi. La nueva especie es única por la presencia de tricomas glutinosos o pegajosos en los lóbulos del cáliz y la superficie externa del ovario ínfero. Basados en los criterios de la UICN, se asigna un estado de conservación preliminar de En Peligro Crítico (CR).

## ﻿Introduction

The flowering plant family Gesneriaceae is in the order Lamiales and comprises 3400+ species in 150+ genera (Weber 2004; Weber et al. 2013). The family is divided into three strongly-supported monophyletic subfamilies (Ogutcen et al. 2021) and seven tribes (Weber et al. 2013, 2020). The majority of New World members are in the subfamily Gesnerioideae and are represented by 1200+ species and 77 genera (Clark et al. 2020). *Monopyle* Moritz ex Benth. & Hook.f. is classified in the tribe Gesnerieae and subtribe Gloxiniinae (Weber et al. 2013, 2020).

*Monopyle* is a genus of terrestrial understory or epiphytic herbs distributed from Guatemala to northern South America. There are 11 described species of *Monopyle* in Ecuador (Keene 2013). The genus currently comprises 22 recognized species (Clark et al. 2020). The addition of *Monopyleglutinosa* brings the total species diversity to 23. The actual number of species is probably double what is currently recognized, based on preliminary estimates from ongoing monographic work by Keene (2013). Additionally, recent exploratory expeditions have yielded numerous new or undescribed species poorly represented in herbaria. Here, we describe a new species that was collected in 2022 during a research expedition to the western Andean foothills.

*Monopyle* is morphologically complex and has had little attention since Morton’s monographic revision (Morton 1945). The genus is traditionally characterized by strongly anisophyllous opposite leaves, campanulate corollas, and the presence of uncinate trichomes (Keene 2013). Additional diagnostic characters that define *Monopyle* include variably swollen internodes, a nodal ridge, and the presence of an osmophore (floral fragrance gland) at the base of the corolla. Many *Monopyle* species are presumably local endemics and appear to be restricted to a specific watershed. Narrow distributions are likely the result of the minute seeds limited by a splash-cup seed dispersal mechanism.

## ﻿Materials and methods

Plants were vouchered and photographed during a 2022 field expedition to Ecuador (Clark 2022). Specimens were deposited at the Pontificia Universidad Católica del Ecuador (**QCA**), Marie Selby Botanical Gardens (**SEL**), United States National Herbarium (**US**), New York Botanical Garden (**NY**), and Missouri Botanical Garden (**MO**). Digital images were taken of live specimens in the field using a Nikon D100 DSLR with a Nikon 105 mm lens and a Nikon SB-29s ring flash. Morphological observations and measurements were made from live collections, alcohol-preserved material, and digital images using the program ImageJ (https://imagej.nih.gov/ij/).

We assessed the extinction risk of *Monopyleglutinosa* following the IUCN Red List Categories and Criteria (2022) and guidelines of the IUCN Standards and Petitions Committee (2022). We considered observations, collection localities and population estimates from fieldwork. Species extent of occurrence (**EOO**) and area of occupancy (**AOO**) were calculated using *GeoCAT* (Bachman et al. 2011; http://geocat.kew.org/) with the default setting of 2 km^2^ grid.

## ﻿Taxonomic treatment

### ﻿ Monopyle glutinosa

Taxon classificationPlantaeLamialesGesneriaceae

J.L.Clark & Keene sp. nov.

415F1577-024F-5A0E-A7C1-20C705857D82

urn:lsid:ipni.org:names:77305891-1

Fig. 1

#### Type.

Ecuador. Cotopaxi: cantón Pujuli, western lowland border of Reserva Ecológica Los Illinizas, trail towards finca of Narcisssa Castellano, trailhead accessed via road Guayacán-Pucayacu, 0°48'44"S, 79°5'37.5"W, 1014 m alt., 11 Mar 2022, *J.L. Clark*, *C. Restrepo & F. Tobar 16489* (holotype: US; isotypes: MO, NY, QCA, SEL).

#### Diagnosis.

Similar to *Monopyleecuadorensis*, differing in larger calyx lobes that reach 1.5 cm in length (*vs.* 0.5–1.0 cm long in *M.ecuadorensis*), larger campanulate corolla tube that exceeds 3.0 cm in length (*vs.* corolla tube less than 3 cm in *M.ecuadorensis*), and a uniformly dark purple corolla tube (*vs.* broad range of corolla tube colors from uniformly white to white suffused with blue in *M.ecuadorensis*).

#### Description.

Terrestrial herb; roots fibrous, shoots dorsiventral, usually light green, occasionally green suffused with red, 20–60 cm tall, 2.5–5 mm diam., glabrous. Leaves opposite, strongly anisophyllous, interstipular scar present; the larger leaf of pair with petioles (4–) 7–17 mm long, uniformly green or green suffused with red, glabrous, blade asymmetrical ovate to elliptic, base oblique, to 10 mm between bases, apex acuminate, (5–) 8.5–23.3 × 3.0–5.8 (–8) cm, subentire to serrate, adaxially light green, sparsely pilose, abaxially green, puberulent to pilose with uncinate trichomes (more so on veins); the smaller leaf of a pair with petioles to 5 mm (some appearing sessile), glabrous, blade ovate to orbicular, base oblique (appearing equilateral), apex acuminate to cuspidate, 0.9–2.4 × 0.5–1.2 cm, entire to serrate towards the apex, adaxially and abaxially similar to larger leaf. Inflorescence a terminal, erect, compound cyme (appearing paniculate); peduncle 5–10 cm, glabrous, bracts in pairs 3–5 × 0.5–1 mm, persistent, opposite, adaxially and abaxially glabrous; rachis to 10 cm long, 3–10 nodes, with 2 cymules per node; pedicel 6–9 mm long. Calyx uniformly light green to uniformly wine red, lobes five, broadly ovate at base and acuminate at apex, 11–14 × 3–5 mm, connate at base, outer surface with dense sticky trichomes, inner surface nearly glabrous. Corolla campanulate, uniformly dark purple, base sometimes white suffused with purple, 30–45 × 15–20 mm, sparsely pilose, minute gland-tipped trichomes on the inner dorsal surface of the tube (inserted above androecium), osmophore present; corolla lobes with minute glandular trichomes along margin of the lobes, lateral and dorsal lobes 7–9 × 4–6 mm, ventral lobe 9–11 × 6–7 mm. Androecium with four stamens, 4–5 mm long, didynamous, included, filaments 3–5 mm long, adnate to corolla, anthers 0.8–1.1 × 0.5–0.7 mm, connivent for up to 1 mm; nectary absent. Gynoecium with inferior ovary, to 2 mm wide, densely pilose with glandular trichomes that extend to the calyx lobes, style to 5.6 mm long, glabrous, stigma stomatomorphic. Fruits not observed.

#### Phenology.

Flowering in March. Fruits not observed.

#### Etymology.

**Figure 1.**
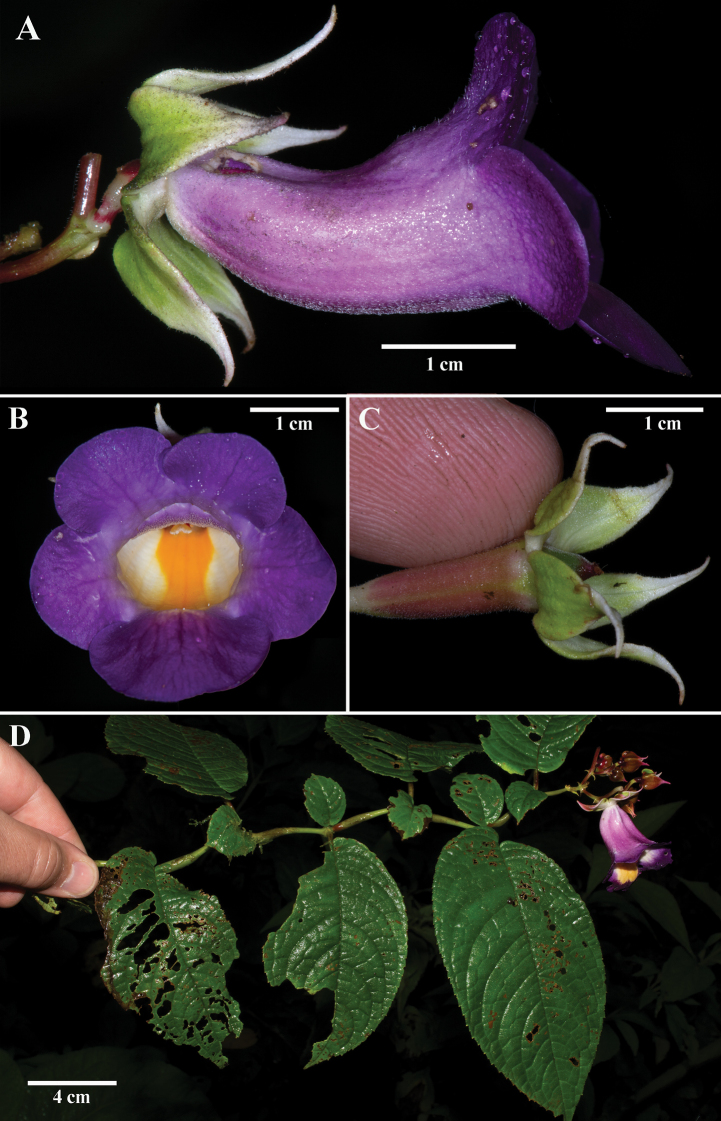
*Monopyleglutinosa* J.L.Clark & Keene **A** lateral view of flower **B** front view of flower **C** inferior ovary and calyx lobes adhering to a finger from the sticky trichomes **D** dorsiventral habit (**A–D** from *J.L. Clark 16489*). Photos by J.L. Clark.

The trichomes on the calyx lobes and inferior ovary allow the flower to cling to an upside-down finger (Fig. 1C). This specific epithet reflects the sticky trichomes on the outer surface of the inferior ovary and calyx lobes.

#### Distribution and preliminary assessment of conservation status.

*Monopyleglutinosa* is endemic to the western Andean slopes of Ecuador. The three known collections are located in the buffer zone and the southern region of Reserva Ecológica Los Illinizas, from disturbed primary forests. GeoCAT calculated the following values for EOO = 46.31 km^2^ and AOO = 12 km^2^. Based on the available information and according to the IUCN Red List Criteria and Guidelines (IUCN 2022; IUCN Standards and Petitions Committee 2022), *M.glutinosa* is preliminarily assessed as Critically Endangered (CR, B1a,biii), based on its limited geographic range (EOO < 100 km^2^) and the uncertain future of habitat conservation of western Andean forests as exemplified by the deforestation for agriculture throughout the buffer zone and inside the park.

#### Comments.

**Figure 2.**
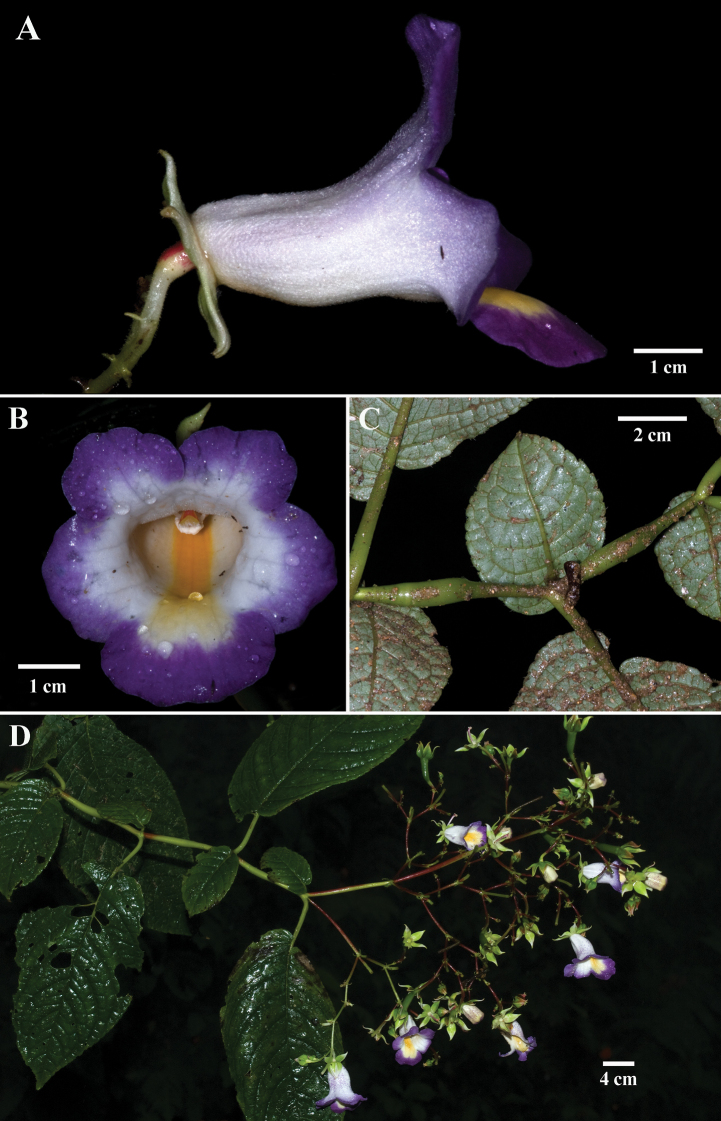
*Monopyleecuadorensis* C.V.Morton **A** lateral view of flower **B** front view of flower **C** swollen region between nodes **D** dorsiventral habit with terminal inflorescence (**A, D** from *J.L. Clark 12301*, **B, C***J.L. Clark 12294*). Photos by J.L. Clark.

*Monopyleglutinosa* differs from all other *Monopyle* by the presence of sticky glandular trichomes intermixed with similarly-sized uncinate trichomes on the outer surface of the inferior ovary and calyx lobes. *Monopyleglutinosa* and *M.ecuadorensis* share a similar terrestrial dorsiventral habit with a terminal inflorescence, swollen regions along the stem between nodes (Fig. 2C), and similar shapes of calyx and corolla. The inflorescence on *M.glutinosa* has shorter peduncles and appears more compact (< 10 cm). The inflorescence on *M.ecuadorensis* has more inflorescence branching and appears broader from longer peduncles, often exceeding 10 cm in length and width (Fig. 2D). The campanulate flowers and broadly ovate calyx lobes are similar to *M.ecuadorensis*. The campanulate corolla tube in *M.glutinosa* exceeds 3 cm (Fig. 1A), in contrast to the smaller corolla tube in *M.ecuadorensis* that rarely exceeds 3 cm in length (Fig. 2A) and 1 cm in width (Fig. 2B). The broadly ovate calyx lobes with acuminate, reflexed apices are longer (ca. 1.5 cm long) in *M.glutinosa* (Fig. 1B) relative to the shorter (ca. 0.5–1.0 cm long) calyx lobes in *M.ecuadorensis* (Fig. 2B). Corolla tube coloration ranges in *M.ecuadorensis* from uniformly white to white suffused with blue (Fig. 2A). In contrast, the corolla tube of *M.glutinosa* is uniformly dark purple (Fig. 1A). *Monopyleecuadorensis* is distributed throughout western Ecuador (usually above 1200 meters). In contrast, *M.glutinosa* is locally endemic and restricted to altitudes below 1200 meters.

#### Additional specimens examined.

Ecuador. **Cotopaxi**: 20 km NW of El Corazón, 19–24 Jun 1967, *B. Sparre 17294* (MO, S); cantón Pujilí, Reserva Ecológica Los Illinizas, sector Paloseco, west of Choasillí, 0°58'34"S, 79°6'58"W, 1700 m alt., 12 Aug 2003, *P. Silverstone-Sopkin et al. 10064* (COL, MO, US).

## Supplementary Material

XML Treatment for Monopyle glutinosa
